# Capturing structural changes of the S_1_ to S_2_ transition of photosystem II using time-resolved serial femtosecond crystallography

**DOI:** 10.1107/S2052252521002177

**Published:** 2021-04-07

**Authors:** Hongjie Li, Yoshiki Nakajima, Takashi Nomura, Michihiro Sugahara, Shinichiro Yonekura, Siu Kit Chan, Takanori Nakane, Takahiro Yamane, Yasufumi Umena, Mamoru Suzuki, Tetsuya Masuda, Taiki Motomura, Hisashi Naitow, Yoshinori Matsuura, Tetsunari Kimura, Kensuke Tono, Shigeki Owada, Yasumasa Joti, Rie Tanaka, Eriko Nango, Fusamichi Akita, Minoru Kubo, So Iwata, Jian-Ren Shen, Michihiro Suga

**Affiliations:** a Research Institute for Interdisciplinary Science and Graduate School of Natural Science and Technology, Okayama University, 3-1-1 Tsushima-naka, Kitaku, Okayama, Okayama 700-8530, Japan; b Graduate School of Life Science, University of Hyogo, 3-2-1 Kouto, Kamigori-cho, Ako-gun, Hyogo 678-1297, Japan; c RIKEN SPring-8 Center, 1-1-1 Kouto, Sayo-cho, Sayo-gun, Hyogo 679-5148, Japan; d Department of Biological Science, Graduate School of Science, The University of Tokyo, 7-3-1 Hongo, Bunkyo-ku, Tokyo 113-0033, Japan; e Institute for Protein Research, Osaka University, 3-2 Yamadaoka, Suita, Osaka 565-0871, Japan; f Division of Food Science and Biotechnology, Graduate School of Agriculture, Kyoto University, Gokasho, Uji, Kyoto 611-0011, Japan; g Department of Chemistry, Graduate School of Science, Kobe University, -1 Rokkodai, Nada-ku, Kobe 657-8501, Japan; h Japan Synchrotron Radiation Research Institute, 1-1-1 Kouto, Sayo-cho, Sayo-gun, Hyogo 679-5198, Japan; i Department of Cell Biology, Graduate School of Medicine, Kyoto University, Yoshidakonoe-cho, Sakyo-ku, Kyoto 606-8501, Japan; j Institute of Multidisciplinary Research for Advanced Materials, Tohoku University, 2-1-1 Katahira, Aoba-ku, Sendai 980-8577, Japan; k Japan Science and Technology Agency, PRESTO, Saitama 332-0012, Japan

**Keywords:** time-resolved serial crystallography, X-ray free-electron lasers, membrane proteins, photosystem II, serial crystallography, molecular movies, protein structures

## Abstract

Photosystem II (PSII) catalyzes light-induced water oxidation through an S*_i_*-state cycle, leading to the generation of di-oxygen, protons and electrons. Pump–probe time-resolved serial femtosecond crystallography (TR-SFX) has been used to capture structural dynamics of light-sensitive proteins. In this approach, it is crucial to avoid light contamination in the samples when analyzing a particular reaction intermediate. Here, a method for determining a condition that avoids light contamination of the PSII microcrystals while minimizing sample consumption in TR-SFX is described. By swapping the pump and probe pulses with a very short delay between them, the structural changes that occur during the S_1_-to-S_2_ transition were examined and a boundary of the excitation region was accurately determined. With the sample flow rate and concomitant illumination conditions determined, the S_2_-state structure of PSII could be analyzed at room temperature, revealing the structural changes that occur during the S_1_-to-S_2_ transition at ambient temperature. Though the structure of the manganese cluster was similar to previous studies, the behaviors of the water molecules in the two channels (O1 and O4 channels) were found to be different. By comparing with the previous studies performed at low temperature or with a different delay time, the possible channels for water inlet and structural changes important for the water-splitting reaction were revealed.

## Abstract 2

A method for determining the sample flow rate and concomitant light condition in time-resolved serial femtosecond crystallography is developed to analyze the intermediate-state structures of photosystem II.

## 1. Introduction   

**Figure 1:**
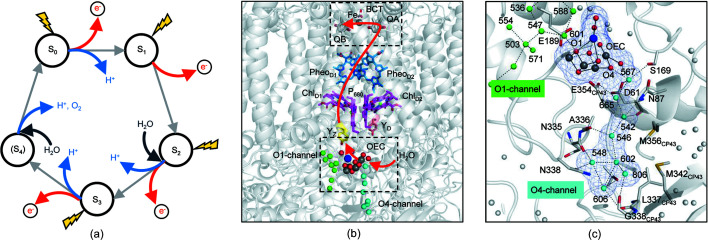
(*a*) S*_i_*-state cycle of the water-oxidation reaction of the OEC. (*b*) Electron-transfer chain of PSII. The flow of electrons is indicated by a red line, and the regions around the OEC, Q_B_ and the non-heme iron are boxed with black dashed lines. (*c*) Enlarged views of the boxed region around the OEC shown in (*b*). Water molecules in the O1 and O4 channels are shown as green and cyan spheres, respectively. The light-blue mesh shows the radius of atoms of the OEC and the water molecules in the O4 channel. The Cl1 and Cl2 channels have been omitted for clarity.

Oxygenic photosynthesis converts light energy into chemical energy, thereby sustaining all aerobic life on Earth. The energy-conversion reaction of photosynthesis is carried out by two photosystems (PSs), PSI and PSII, and both are large membrane-embedded protein–pigment complexes existing on the thylakoid membranes of plants and various algae. PSII uses light energy to extract electrons and protons from water molecules, leading to the oxidation of water and the release of di­oxy­gen as a byproduct. This water-oxidation reaction is catalyzed by the oxygen-evolving complex (OEC) of PSII and proceeds through a light-driven, five-step S*_i_*-state cycle (*i* = 0–4) (Kok *et al.*, 1970 ▸; Shen, 2015 ▸; Cox *et al.*, 2020 ▸) [Fig. 1 ▸(*a*)]. In this S*_i_*-state cycle, S_0_ is the ground state, and the OEC progresses to higher S*_i_* states upon its oxidation by Y_Z_^+^, a tyrosine cation residue generated by P_680_^+^, the photoexcited PSII reaction center P_680_ [Fig. 1 ▸(*b*)]. The release of di­oxy­gen occurs during the S_3_-to-(S_4_)-to-S_0_ transition. The OEC is dark-stable in the S_1_ state, with the chemical composition Mn_4_CaO_5_ (Umena *et al.*, 2011 ▸; Suga *et al.*, 2015 ▸). The OEC changes to an Mn_4_CaO_6_ cluster in the highest metastable S_3_ state by incorporation of a new oxygen atom (O6) near the unique μ-oxo bridge O5, which was first identified at 2.35 Å resolution (Suga *et al.*, 2017 ▸), and further analyzed at resolutions of 2.15 Å (Suga *et al.*, 2019 ▸) and 2.09 Å [the new oxygen atom was referred to as Ox (Ibrahim *et al.*, 2020 ▸)]. The interatomic distance between O6 and O5 has been reported to be 1.9 Å (Suga *et al.*, 2019 ▸) or 2.2 Å (Ibrahim *et al.*, 2020 ▸), which are suitable for an oxyl/oxo type coupling to form di­oxy­gen between them. The detailed mechanism of the water oxidation, however, has not been well understood, in particular concerning the origin of O6 and the proton exit pathways.

The Mn_4_CaO_5_ cluster is embedded inside the protein matrix of PSII and covered by a large area of hydro­philic protein regions in the lumenal side of the thylakoid membrane. Channels for the inlet of substrate waters and egress of the product protons are important for the water-splitting reaction to proceed appropriately. The high-resolution structure of PSII showed multiple hydrogen-bonded networks connecting the site of the Mn_4_CaO_5_ cluster to the lumenal surface of PSII (Umena *et al.*, 2011 ▸; Shen, 2015 ▸; Suga *et al.*, 2015 ▸). These channels may therefore function to allow water into the catalytic site or protons to be transported to the lumen. Four main such channels have been identified: the O1 channel, O4 channel, Cl1 channel and Cl2 channel (Fig. 1 ▸). The O1 and O4 channels are so-called because they start from the oxo-bridges O1 and O4 of the Mn_4_CaO_5_ cluster, respectively, whereas the Cl1 and Cl2 channels are mediated by the Cl1 and Cl2 ions in the vicinity of the Mn_4_CaO_5_ cluster, respectively. It is not clear which of these channels functions in the water inlet or proton egress, and in the latter case, in which S-state transition.

Pump–probe, time-resolved serial femtosecond X-ray crystallography (TR-SFX) using X-ray free-electron lasers (XFEL) is a powerful method to visualize structural dynamics of light-sensitive proteins (Tenboer *et al.*, 2014 ▸; Barends *et al.*, 2015 ▸; Nango *et al.*, 2016 ▸; Pande *et al.*, 2016 ▸; Nogly *et al.*, 2018 ▸; Suga *et al.*, 2020 ▸), including PSII in different S*_i_* states at ambient temperature (Kern *et al.*, 2013 ▸, 2014 ▸, 2018 ▸; Kupitz *et al.*, 2014 ▸; Young *et al.*, 2016 ▸; Suga *et al.*, 2017 ▸; Ibrahim *et al.*, 2020 ▸) or cryogenic temperature (Suga *et al.*, 2019 ▸). XFELs provide femtosecond pulses of X-rays with an approximately billion-fold increase in peak brilliance when compared with conventional synchrotron X-rays, thus enabling collection of diffraction data before the onset of radiation damage (Neutze *et al.*, 2000 ▸). For capturing the intermediate S_*i*_ states of PSII with this method, a flow of PSII microcrystals either in solution or embedded in a matrix is illuminated by a desired number of pump flashes to generate the higher S*_i_* states (one, two or three flashes generate S_2_, S_3_ or S_0_ states, respectively), followed by detection with an XFEL pulse with a temporal delay time after the flash illumination. One of the most critical factors for the success of this experiment is the selection of an optimal light excitation condition (intervals, the boundary of excitation region and power of the excitation laser *etc*.) under particular sample delivery conditions (crystal size, flow diameter, flow rate, overall sample consumption *etc*.). While the light illumination area is primarily defined by the laser spot (Kovacs *et al.*, 2019 ▸; Grünbein *et al.*, 2020 ▸), some reports claimed that the light may scatter to a larger area than its illumination spot (Nogly *et al.*, 2018 ▸). A generally applicable way to determine an effective light excitation condition is adding a generous safety margin in the sample area to be excited and examining the structural changes by TR-SFX under a suitable size and power of the pump laser illumination. However, in the case of enzymes such as PSII that requires multi-flash excitations for the higher S-states, the application of this method is not straightforward under the continuous sample flow condition. The sample area that can be used for the excitation and X-ray diffraction must be well aligned spatially, and the pump/probe illumination area and the sample must be aligned temporally. However, a larger separation between two consecutive flashes may cause the illuminated sample to escape from the area under irradiation by the XFEL pulses. In such cases, higher S_*i*_ states may not be captured by this TR-SFX method, and other methods such as fixed-target crystallography (Suga *et al.*, 2019 ▸) or a tape drive (Ibrahim *et al.*, 2020 ▸) may be potentially applicable.

In the present study, we developed a method to determine an optimal light illumination condition for successful TR-SFX to analyze the structures of the intermediate S_*i*_ states of PSII. By altering the flash interval distances with maximum delay time and examining the structural changes that occur during the S_1_-to-S_2_ transition, a boundary of the excitation region was determined. Based on the light illumination conditions determined, we analyzed the PSII structure in the S_2_ state at 2.4 Å resolution. Structural changes were found in the OEC, the O1 and O4 channels, and the Q_B_-binding site, providing important insights into the substrate water delivery during the water-oxidation reaction.

## 2. Methods   

### 2.1. Preparation and crystallization of photosystem II   

Highly active dimeric PSII core complexes were purified from the thermophilic cyano­bacterium *Thermosynechococcus vulcanus* cells and crystallized as described previously (Shen & Kamiya, 2000 ▸; Umena *et al.*, 2011 ▸; Suga *et al.*, 2017 ▸). The microcrystals grew to a maximum size of 100 µm. They showed good quality in diffraction as well as high efficiency in the progress of the S*_i_*-state cycle (Kato *et al.*, 2018 ▸). Before the TR-SFX experiments, the microcrystals were pre-flashed by an Nd:YAG laser at 532 nm with a diameter of 7 mm at an energy of around 52 mJ cm^−1^ (Suga *et al.*, 2017 ▸) to oxidize the tyrosine D residue (D2-Y160) and decrease the contamination of the S_0_ state. The microcrystals were dehydrated with a buffer containing 20% glycerol, 10% PEG 1450, 10% PEG 5000 MME, 2% di­methyl sulfoxide (DMSO) and 10 m*M* potassium ferricyanide by stepwise replacement of the solution, which took around 2 h. Following the dehydration, the crystals were mixed with a silicon grease (Sugahara *et al.*, 2015 ▸) and used for the TR-SFX experiments. All the procedures, including purification, crystallization, pre-flash illumination, dehydration and mixing with the grease matrix were performed in dark- or dim-green light.

### 2.2. Diffraction experiments at SACLA-XFEL   

The PSII crystals mixed with the grease matrix were loaded into an injector (Shimazu *et al.*, 2019 ▸) with a nozzle diameter of 140 µm and set in a pump–probe system based on Diverse Application Platform for Hard X-ray Diffraction (DAPHNIS) in the SPring-8 Ångstrom Compact Free Electron Laser (SACLA) (Tono *et al.*, 2015 ▸; Kubo *et al.*, 2017 ▸). The flow rates were as follows: 2.5 µl min^−1^ for the dark datasets; 4.9, 7.3, 8.5 and 9.8 µl min^−1^ for each ‘light’ dataset at a delay time of −50 ns (that is, 50 ns before the laser flash); 9.8 µl min^−1^ for the light dataset at a delay time of 10 ms. The actual speed of the stream (the distance traveled by the sample per unit time) under the volumetric flow rate specified was examined at the preliminary stages of the experiment. We monitored the stream of the PSII microcrystals using a high-speed camera at a flow rate of 7.8 µl min^−1^ with a nozzle diameter of 125 µm. Under this condition, the speed of the stream was expected to be 10.6 mm s^−1^, whereas the actual speed and diameter of the stream were 9.7 ± 0.9 mm s^−1^ and 131 ± 7 µm, respectively. When the stream was stable, the actual speed was constant. This was also confirmed with microcrystals in the lipid cubic phase (Nango *et al.*, 2019 ▸). Although we did not monitor the speed of the stream throughout the experiment, we collected diffraction images only when the stream was stable, and we stopped data collection when the stream showed a stop/go behavior or balled up for a limited amount of time, or wiggled *etc*.

As the pump laser, a 532 nm pulse from an Nd:YAG laser source (Minilite-I, Continuum) with a repetition of 10 Hz, was split into two beams, and each beam was focused on the microcrystals from two different directions with an angle of 160° for efficient excitation (Suga *et al.*, 2017 ▸). The pump focal diameter was set to 240 µm at the targeted sample position, and its energy was 42 mJ cm^−2^ from each direction. Diffraction images were collected using femtosecond X-ray pulses from SACLA at BL3 with the following pulse parameters: 2–10 fs pulse duration, 7 keV X-ray energy, 0.5% (FWHM) energy bandwidth, pulse flux: ∼7 × 10^10^ photons per pulse, 3.0 (*H*) × 3.0 µm (*W*) beam size, 30 Hz repetition rate. The XFEL pulses were provided to the pump focal center either before 50 ns of the pump laser pulse for the ‘light’ datasets at different flow rates, or 106 µm downstream from the pump focal center after 10 ms of the pump laser pulse for the light dataset, which fully transforms the S state to the S_2_ state. Diffraction images were recorded by a multiport CCD detector. Because the excitation laser pulses were provided at 10 Hz and the XFEL pulses had a repetition rate of 30 Hz, all ‘pump-on’ images were recorded at 10 Hz for the ‘light’ datasets, whereas the diffraction data for the dark dataset were recorded at 30 Hz in a separate run.

### 2.3. Data processing   

The diffraction data collected at SACLA were monitored by the program *Cheetah* (Barty *et al.*, 2014 ▸; Nakane *et al.*, 2016 ▸), and the diffraction images passing through the ‘filter’ with pre-defined thresholds of diffraction spot numbers (recorded as ‘hits’) were used for the subsequent processing. Indexing, integration, scaling and merging of the images were carried out by programs in the *cctbx.xfel* suite (Sauter *et al.*, 2013 ▸; Sauter, 2015 ▸; Waterman *et al.*, 2016 ▸). The diffraction images were indexed and integrated by the program *dials.stills_process* using the geometry of the detector and a camera distance refined by the program *cspad.cbf_metrology* (Brewster *et al.*, 2018 ▸). The unit-cell parameters (*a* = 126.3, *b* = 232.1, *c* = 289.0 Å, and α = β = γ = 90°), originally determined by the program *CrystFEL* (White *et al.*, 2012 ▸) were provided during the indexing process. The number of indexed images for each dataset were as follows: 96459 for dark1; 41071 for dark2; 18216 for −50 ns ‘light’ at 4.9 µl min^−1^; 13858 for −50 ns ‘light’ at 7.3 µl min^−1^; 20449 for −50 ns ‘light’ at 8.5 µl min^−1^; 22419 for −50 ns ‘light’ at 9.8 µl min^−1^; 17247 for 10 ms light at 9.8 µl min^−1^ (Table 1 ▸). Integrated images were merged by the program *cxi.merge* with the post-refinement rs2 algorithm, and a filter based on the value of *I*/σ(*I*) was not applied in order to include weak signals at high resolutions. Dataset dark1 or dark2 could be used as the reference model in the processing of ‘light’ datasets from the same purification batch of samples. All datasets were processed to 2.25–2.40 Å resolutions based on the criteria of CC_1/2_ < 50%. The *R*_iso_ values between the datasets were 0.073–0.100 in the same sample batch, and 0.095–0.112 between the different sample batches. Because different sample batches gave a slightly higher *R*_iso_ between the different datasets, and because a lower *R*_iso_ was crucial to detect meaningful structural changes based on the isomorphous difference Fourier map, we collected the two different dark datasets (dark1 and dark2) independently for the different sample batches used.

### 2.4. Model building and map calculation   

The initial phase of the dark dataset was obtained by molecular replacement with the program *Phaser-MR* in the *CCP4* package (Collaborative Computational Project, 1994 ▸), using the previous PSII structure at room temperature (PDB entry 5ws5; Suga *et al.*, 2017 ▸) as the search model. Then, an initial rigid-body refinement, followed by refinement of coordinates, *B* factors and TLS, was performed using the program *Phenix* (Adams *et al.*, 2010 ▸; Afonine *et al.*, 2012 ▸) combined with manual modifications of the program *Coot* (Emsley & Cowtan, 2004 ▸). The restraints used here were the same as those used previously (Suga *et al.*, 2017 ▸, 2019 ▸). The final *R*_work_ and *R*_free_ were 0.169 and 0.211 for the dark1 dataset, and 0.160 and 0.208 for the dark2 dataset (Table 1 ▸). The phases from the refined model of dark1 or dark2 were used to calculate the isomorphous difference Fourier maps between dark and each −50 ns, ‘light’ dataset, or 10 ms, light dataset. Strong peaks in the isomorphous difference Fourier maps were found around the regions of the OEC, Q_B_ and non-heme iron, and these regions were built as a mixture of dominant S_2_ and minor S_1_ states based on the transition efficiency of the PSII microcrystals (Kato *et al.*, 2018 ▸). We first refined the coordinates of the dominant S_2_ state only with fixed *B* factors. Then we refined the *B* factors of atoms in the regions mentioned above which show structural changes during the S_1_-to-S_2_ transition by assuming the populations of the S_2_ and S_1_ states at (0.9, 0.1), (0.8, 0.2), (0.7, 0.3) and (0.6, 0.4), and found that the residual electron densities and *B* factors after the refinement were reasonable for the populations of S_2_ and S_1_ states of 0.7 and 0.3, respectively. The determined occupancy was consistent with the transition efficiency of the PSII microcrystals (Kato *et al.*, 2018 ▸). Thus, this occupancy was used to refine the final structure in the S_2_ state. This includes 29 waters, 42 residues, 2 OECs, 2 Q_B_s, 2 BCTs and 2 non-heme irons. The statistics for structural refinement are provided in Table 1 ▸.

## 3. Results   

### 3.1. Determination of a boundary of the excitation region using TR-SFX   

**Figure 2:**
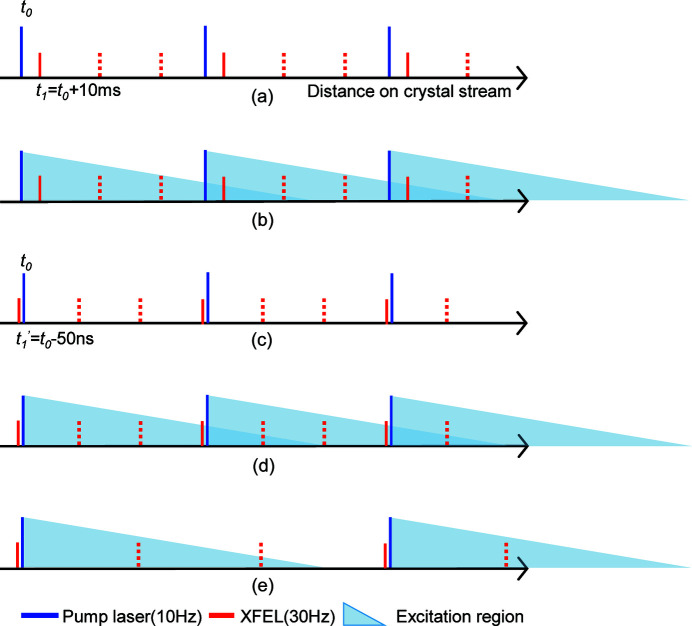
Relative timing of the pump lasers and XFEL pulses with Δ*t* = 10 ms (*a*) and Δ*t* = −50 ns (*c*). Schematic representations of the boundaries where the pump lasers reaching a slower flow rate with Δ*t* = 10 ms (*b*) or Δ*t* = −50 ns (*d*), and at a faster flow rate with Δ*t* = −50 ns (*e*). Note that the region exposed to XFEL pulses interacts with multiple pump lasers (*b*) or an unintended laser (*d*) when a slower flow rate was employed. XFEL pulses that were not recorded for the ‘light’ datasets are shown as dashed lines. The excitation region is shown as a triangle for clarity, but it is not a linear profile in the experiment.

We performed TR-SFX as described previously (Suga *et al.*, 2017 ▸; Sugahara *et al.*, 2015 ▸). In this approach, PSII microcrystals were mixed with a grease matrix and ejected from a micro-extrusion injector. The flow of the PSII microcrystals was excited by a single flash to advance the S state to S_2_. Fig. 2 ▸(*a*) shows a scheme representing the interaction between the pump excitation region and the XFEL pulse in the TR-SFX experiment with a delay time (Δ*t*) of 10 ms at repetition rates of 10 and 30 Hz for the pump and XFEL, respectively. It should be noted that, although the pump beam was focused on the sample stream with a top-hat shape (ϕ = 250 µm) (Kubo *et al.*, 2017 ▸), the effective excitation region extended upstream and downstream possibly due to the pump-light scattering on the stream as well as tailing of the laser illumination area. Thus, the excitation region is schematically depicted by a triangle in the figure, indicating a gradual decrease in the pump photon density along the sample stream. When a flow rate is not fast enough for the sample exchange, the pump excitation region interacts with the next pump-XFEL pulse [Fig. 2 ▸(*b*)]. To avoid such erroneous light-contamination, we designed a test experiment with Δ*t* = −50 ns, where the negative time points signify that the X-rays arrive 50 ns before the laser, so that the delay to the next laser pulse (at 10 Hz) is 99.99995 ms for the ‘pump-on’ data [Fig. 2 ▸(*c*)]. As the XFEL was operated at 30 Hz, there will also be ‘pump-off’ data between the two ‘pump-on’ data [red dashes in Fig. 2 ▸(*c*)], which were not processed in the present study. On this −Δ*t* condition, if the flow rate is slow, the microcrystals would be illuminated partially by the preceding flash, resulting in ‘one-flash’ illumination [Fig. 2 ▸(*d*)]. However, at a sufficiently fast flow rate, the microcrystals at the position of the XFEL pulse will escape from the preceding flash illumination [Fig. 2 ▸(*e*)], resulting in a ‘dark dataset.’ Accordingly, we can check light contamination, including a possible effect of pump light scattering on the sample stream, under the given experimental conditions (pump-illumination size and intensity, sample flow rate *etc*.).

Four diffraction datasets were collected at different flow rates of 4.9, 7.3, 8.5 and 9.8 µl min^−1^ (corresponding to 2.0 times, 3.0 times, 3.5 times and 4.0 times that for the dark datasets, respectively). In addition to these four datasets, we collected two independent dark datasets by different preparations (dark1 for the light-illuminated, flow rate 4.9 and 7.3 µl min^−1^ experiments, and dark2 for the light-illuminated, flow rate 8.5 and 9.8 µl min^−1^ experiments) at a flow rate of 2.5 µl min^−1^, and a light dataset with Δ*t* = 10 ms at a flow rate of 9.8 µl min^−1^. All datasets were processed at 2.25 to 2.40 Å resolutions (Table 1 ▸).

**Figure 3:**
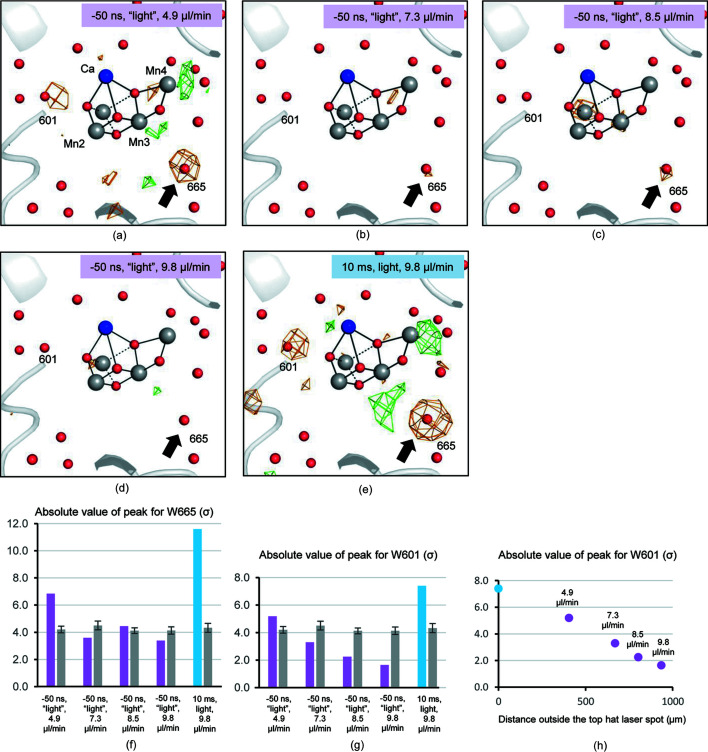
(*a*)–(*e*) S_1_-state structure superimposed with the *F*_obs_(light) − *F*_obs_(dark) isomorphous difference Fourier map contoured at +4.0σ (green) and −4.0σ (orange) calculated with the ‘light’ datasets obtained under the conditions (*a*) −50 ns, ‘light’, 4.9 µl min^−1^ flow rate; (*b*) −50 ns, ‘light’, 7.3 µl min^−1^; (*c*) −50 ns, ‘light’, 8.5 µl min^−1^, (*d*) −50 ns, ‘light’, 9.8 µl min^−1^; and (*e*) 10 ms, light, 9.8 µl min^−1^. The changes in W665 were indicated by black arrows. Average peak heights of the difference map at the position of (*f*) W665 and (*g*) W601 calculated from two PSII monomers and maximum noises or systematic errors. Maximum noise or systematic errors with error bars were calculated from the five strongest noise peaks observed outside the PSII protein complex and are shown in gray. (*h*) Peak heights at the position of W601 were plotted against the distance the sample traveled outside the top-hat laser spot where the sample is no longer directly illuminated. The 10 ms, light, 9.8 µl min^−1^ dataset corresponds to distance zero (the focus of the laser beam), and for the other datasets with a delay time of −50 ns, the calculated total distance traveled −125 µm was used.

We evaluated the boundary of the excitation region as follows. Isomorphous difference Fourier maps between the ‘light’-illuminated and dark datasets were calculated with the phases obtained by the refinement of the dark datasets, which showed a negative peak covering W665, the second water molecule from O4 in the O4 channel [Fig. 1 ▸(*c*)]. Since a similar negative peak has been observed in the previous studies, indicating that W665 becomes highly disordered during the S_1_-to-S_2_ transition (Suga *et al.*, 2019 ▸; Kern *et al.*, 2018 ▸), we take the height of the Fourier difference peak of W665 as an indicator for the light-induced structural changes. The *F*_obs_ (−50 ns, ‘light’ at 4.9 µl min^−1^) minus *F*_obs_ (dark) difference Fourier map showed a peak height of −6.9σ at the position of W665 [Fig. 3 ▸(*a*), Table S1 of the supporting information]. This peak height is lower than that observed with a delay time of 10 ms after the excitation flash [Fig. 3 ▸(*e*)], but is higher than the maximum noise level or systematic errors [Fig. 3 ▸(*f*), Table S1], suggesting that, at this flow rate, the microcrystals at the target position of the XFEL shot have been excited by the preceding flash illumination. Thus, the flow rate of 4.9 µl min^−1^ is too slow to avoid light-contamination by the preceding flash in the excitation region. The average height of the light-minus-dark Fourier difference peak of W665 in two non-crystallographic symmetry-related PSII monomers was reduced when the flow rate was increased [Figs. 3 ▸(*b*)–3(*d*), Table S1], and reached a level not visible in the difference map contoured at ±4.0σ at a flow rate of 9.8 µl min^−1^ [Fig. 3 ▸(*d*)], which is also well below the maximum noise level or systematic errors [Fig. 3 ▸(*f*)]. This indicates that at the flow rate 9.8 µl min^−1^, no apparent light-minus-dark Fourier difference peak was observed. Unexpectedly, the peak at the 8.5 µl min^−1^ flow rate (−4.5σ) was slightly higher than that of 7.3 µl min^−1^ (−3.6σ) (Table S1). So we also compared the peak heights of W601 (another water molecule that disappeared in the S_1_-to-S_2_ transition, discussed in more detail below) and confirmed that the signal decreased consistently [Fig. 3 ▸(*g*), Table S1]. These results suggest that the variations in the peak heights of W665, as well as many other changes (data not shown) in the structure derived from data collected at flow rates of 7.3 and 8.5 µl min^−1^ may originate from non-isomorphism between the different crystal batches.

**Figure 4:**
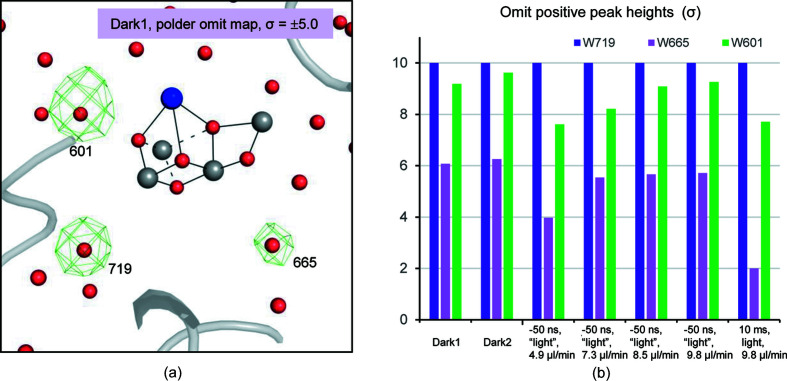
(*a*) S_1_-state structure superposed with the omit map for W601, W665 and W719 calculated from the dark1 dataset contoured at +5.0σ (green) and −5.0σ (orange). (*b*) Positive peak heights of the omit map determined for the water molecules. W719 is a well defined water molecule that does not change its structure during the S-state cycle, thus its positive omit density was used as a baseline. The figure shows relative heights of omit maps of water molecules based on that of W719.

The excitation region was further estimated based on difference densities of water molecules, as the nozzle diameter used to collect the data was 140 µm, which was different from that of 125 µm used for the off-line monitoring of the flow rate (see Methods[Sec sec2]), suggesting an error of about 10% in the real flow rate when compared with that calculated. When the peak height of W601 was plotted with respect to the distance the sample traveled outside the top-hat laser spot where the sample is no longer directly illuminated, a high excitation yield was observed even at 405 µm, but was diminished at 935 µm [Fig. 3 ▸(*h*)]. Because these changes are based on the isomorphous differences which are readily affected by non-isomorphism between datasets, we examined the changes based on the *F*_obs_ − *F*_calc_ omit maps. Three water molecules, W665, W601 and W719 were omitted in both PSII monomers of the dimer and the omit electron densities were scaled to the dark1 dataset [Fig. 4 ▸(*b*)]. Here, W719 is a well defined molecule that does not change its position upon light illumination, and therefore its positive omit electron density is used as a baseline for comparison to determine the peak heights. When compared with the dark1 and dark2 datasets, electron densities for W665 and W601 became lower at flow rates of 4.9 and 7.3 µl min^−1^, but were comparable at flow rates of 8.5 and 9.8 µl min^−1^ (Fig. 4 ▸). Therefore, we concluded that the flow rate 9.8 µl min^−1^ gives rise to no light contamination at the position of the XFEL shot. We collected the light-illuminated dataset with Δ*t* = 10 ms after the flash illumination at this flow rate to analyze the PSII structure in the S_2_ state.

### 3.2. Structural determination of PSII in the S_2_ state   

**Figure 5:**
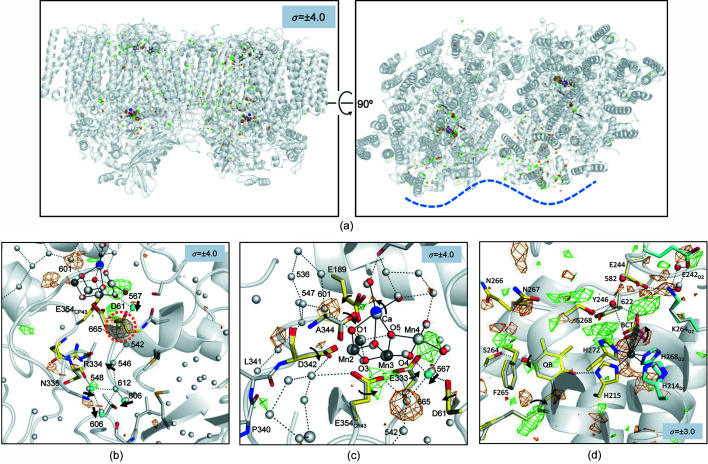
Structures of PSII in the S_1_ (gray) and S_2_ states (colored) superimposed with an isomorphous difference Fourier map showing (*a*) the overall PSII structure, and the regions of the (*b*) O4 channel, (*c*) OEC and (*d*) Q_B_ site. The difference Fourier map was contoured at ±4σ (*a*)–(*c*) and ±3σ (*d*) in the same color as those in Fig. 3 ▸. The surface area where the minor structural changes are distributed is indicated by a blue dashed line. Hydrogen-bonded networks of water molecules are represented by black dotted lines, and the structural changes are indicated by black arrows. W665 is encircled with a red dashed line in (*b*).

The *F*_obs_ (10 ms, light, 9.8 µl min^−1^) − *F*_obs_ (dark) isomorphous difference Fourier map showed a strong signal with a peak height above −11.6σ at the position of W665, suggesting that the microcrystals were successfully excited to progress to the S_2_ state [Figs. 5 ▸(*a*)–5(*c*)]. Many peaks can be seen in the Fourier difference map when we decrease the contour level below ±3σ; thus we consider that the average noise level is around ±3σ, and peaks above ±3σ may represent real structural changes induced by one-flash illumination. We observe many peaks above ±4.5σ that are centered around the OEC and the electron-transfer chain, and are consistent with previously observed structural changes that occur during the S_1_-to-S_2_ transition. They are distributed around the OEC, bicarbonate (BCT), non-heme iron and Q_B_, and are observed similarly in both PSII monomers. For these reasons they were interpreted as light-induced structural changes. However, some weaker peaks at around ±4σ were found at one side of the monomer–monomer interface [Fig. 5 ▸(*a*)]. As the isomorphism between the two datasets was relatively low, these weaker peaks may not be related to the light-induced structural changes. Instead, these may arise from differences in the batches of samples used, since it was difficult to control the sample purification conditions and sample states, such as dehydration of the crystals, to be entirely uniform.

**Figure 6:**
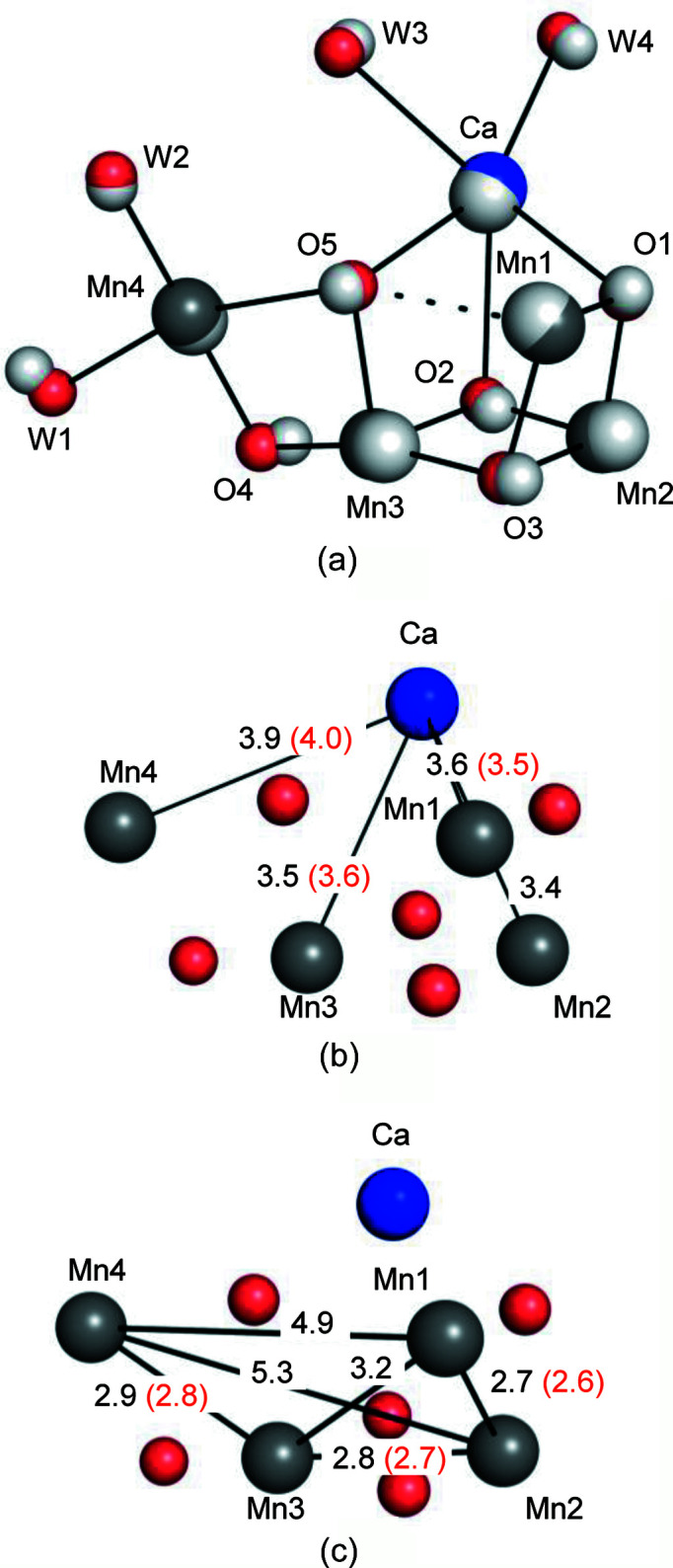
(*a*)–(*c*) OEC structures in the S_1_ and S_2_ states are shown as gray and colored atoms, respectively. Color codes: blue for calcium; cyan for manganese; red for oxygen. The color codes for the OEC are the same in all figures unless otherwise noted. In (*b*) and (*c*), interatomic distances are given in ångstroms, with the numbers in black for S_1_ and red for S_2_. The distances in the S_2_ state were shown only when they were greater by more than 0.1 Å compared with the corresponding distances in the S_1_ state.

We refined the 10 ms light dataset as a mixture model consisting of 70% S_2_ state and 30% S_1_ state for the Mn_4_CaO_5_ cluster, its nearby residues, the region around Q_B_ and the BCT binding site. These include 29 waters, 42 residues, 2 OECs, 2 Q_B_s, 2 BCTs and 2 non-heme irons. This gives rise to equivalent values of the temperature factors between the two equivalent atoms in the multiple model, and this population of S_2_ and S_1_ states after one-flash illumination is similar to the efficiencies of the S*_i_* state transition estimated by Fourier transform infrared spectroscopy (Kato *et al.*, 2018 ▸; Suga *et al.*, 2017 ▸). The OEC structures in the S_1_ and S_2_ states determined in the present study are similar to those reported in previous studies (Suga *et al.*, 2019 ▸; Ibrahim *et al.*, 2020 ▸). The OEC structure in the S_2_ state was in the open-cubane form, in which the right side of the O5 is open, giving rise to the five-coordinate trigonal bipyramidal coordination of Mn1. All changes in the Mn–Mn and Mn–Ca distances during the S_1_-to-S_2_ transition were less than the error range of the coordinates at the current resolution (Fig. 6 ▸). However, the changes found in the isomorphous difference Fourier map, such as the shortening of Mn3–Mn4 and elongation of Mn1–Mn3, Mn3–Ca and Mn4–Ca, were consistent with the previous study with the diffraction data collected at 100 K for the room-temperature-trapped S_2_ state (Suga *et al.*, 2019 ▸) (Fig. 6 ▸ and Table 2 ▸). In association with the movements of these manganese atoms, some ligand residues of the OEC (D1-E189, E333, D342, A344 and CP43-E354) also moved slightly, as previously observed (Suga *et al.*, 2019 ▸) [Fig. 5 ▸(*c*)].

### 3.3. Structural changes in the O4 and O1 channels   

Among the four channels, the O4 channel has been suggested to function as the pathway of proton release during the S_0_-to-S_1_ transition based on theoretical calculations (Saito *et al.*, 2015 ▸). On the other hand, other groups have argued that it serves as the source of substrate water by a ‘pivot’ or ‘carousel’ mechanism in the S_2_-to-S_3_ transition (Wang *et al.*, 2017 ▸; Retegan *et al.*, 2016 ▸; Kawashima *et al.*, 2018 ▸). As described above, upon transition to the S_2_ state, W665 in the O4 channel becomes highly disordered. This is accompanied by slight shifts of the nearby residues D1–D61 and CP43-E354 toward the position of W665, resulting in a narrowing of the space that has been occupied by W665 [Figs. 3 ▸(*e*) and 5 ▸(*b*)]. A water cluster (W546, W548, W612, W606 and W806) leading to the lumenal surface in the O4 channel also shifted its position during the S_1_-to-S_2_ transition, and the shift of W548 induced structural changes of its hydrogen-bond partners D1-R334 and D1-N335 [Fig. 5 ▸(*b*)]. These changes in the O4 channel were similar to a previous study at cryogenic temperature (Suga *et al.*, 2019 ▸). However, another water molecule, W757, connected to W548 and W606 in this channel, was found to become disordered in the S_1_-to-S_2_ transition at cryogenic temperature (Suga *et al.*, 2019 ▸), but this water molecule was not detected in either the S_1_ or S_2_ structures in the present study. Presumably, due to its peripheral location and thus its weaker association within the channel, W757 probably possesses higher mobility at room temperature, at which the TR-SFX experiments were conducted in the present study.

Another noticeable change observed near the OEC was a negative peak of −7.4σ covering W601, the hydrogen-bond donor to O1 and one of the members of a diamond-shaped water cluster in the O1 channel [Figs. 5 ▸(*b*) and 5(*c*)]. The negative peak of W601 has a slight displacement towards the O1 atom of OEC in the S_2_-state structure compared with its position in the dark structure. This indicates that W601 became disordered and shifted in the S_1_-to-S_2_ transition. This change was not found in our previous study performed at cryogenic temperature (Suga *et al.*, 2019 ▸). Another SFX study at ambient temperature by Kern *et al.* (2018 ▸) reported shifts of three water molecules W601, W547 and W536 (W26, W27 and W30) of the diamond-shaped water cluster. In contrast, our previous fixed-target SFX study at cryogenic temperature reported that W571, a water molecule found at a cryogenic temperature only, became disordered instead of W601 (Suga *et al.*, 2019 ▸). Though we could not exclude the possibility of difference in the solution conditions under which the crystals were prepared and/or kept, or temperature employed (ambient versus cryogenic), these differences may indicate high mobility of the water molecules in the O1 channel during the S_1_-to-S_2_ transition.

### 3.4. Q_B_ and the non-heme iron site   

After one-flash illumination, the second bound quinone electron acceptor Q_B_ undergoes a reduction to form a stable plastosemi­quinone intermediate Q_B_^−^. The isomorphous difference Fourier map showed a positive peak covering the Q_B_ head and a pair of positive and negative peaks over the Q_B_ tail, suggesting its slight movement during the S_1_-to-S_2_ transition [Fig. 5 ▸(*d*)]. It should be noted that the peaks around the Q_B_ site were smaller when compared with the OEC, or the O4 and O1 channels. Therefore, the motion of the residues discussed here can be confined to those showing a positional shift in coordinates and a pair of positive–negative difference densities. The *B* factor of the Q_B_ head was decreased from 123 Å^2^ in the S_1_ state to 101 Å^2^ in the S_2_ state, and the features of the isomorphous difference Fourier map suggest a shortening of the hydrogen-bond interaction between the head of Q_B_ and D1-S264, indicating a tighter binding of the semi­quinone Q_B_^−^ to D1-S264. Thus, the first protonation of Q_B_ may occur at this site. This notion is consistent with the theoretical calculation that the first proton transfer from D1-S264 to Q_B_ is an energetically downhill process, whereas it is an uphill one from H215 to Q_B_ (Saito *et al.*, 2013 ▸). Several pairs of positive and negative peaks were also found around the hydrogen-bonded network formed by BCT, D1-Y246, D1-E244, D2-K264 and D2-E242, and BCT moved 0.26 Å away from the non-heme iron [Fig. 5 ▸(*d*)]. These changes may be related to the reduction of the non-heme iron or proton uptake after one-flash illumination, which in turn suggests that these residues and BCT form part of the proton inlet channel for the protonation of Q_B_^−^.

## 4. Discussion   

For the successful pump–probe TR-SFX experiments to analyze intermediate structures of proteins using light as the pump to initiate the reaction, it is critical to find an optimal light excitation condition with an optimal sample delivery system. These conditions vary considerably depending on the sample of interest as well as the setup of the pump–probe SFX system. Under optimal conditions, one can save a large amount of valuable protein sample as well as XFEL beam time for obtaining structures of the targeted intermediate states. In the present study, we showed a method to determine a boundary excited by the pump by altering the flow rate of the microcrystals and evaluating the resulting light-induced structural changes of PSII. It was shown that the optical illumination area of the laser is scattered into an area well beyond that defined by the laser spot itself, and the flow rate of the crystal stream has to be fast enough to avoid light contamination by the preceding flash illumination. This calls for caution in defining the optical illumination conditions in TR-SFX experiments (Nogly *et al.*, 2018 ▸; Kovacs *et al.*, 2019 ▸; Grünbein *et al.*, 2020 ▸). With the optimal conditions obtained, we analyzed the structure of PSII in the S_2_ state at room temperature by the TR-SFX method. This method can be applied to other samples, whose catalytic reaction is triggered by light, using systems which deliver microcrystals in a continuous flow. Importantly, this method also provides suitable light conditions for samples, such as PSII, that require successive flash excitations for the catalytic cycle.

The PSII structures in the S_1_ and S_2_ states, as well as the isomorphous difference Fourier map obtained in the present study, revealed the light-induced structural changes localized at the OEC, Q_B_ site and water molecules in the O1 and O4 channels. Comparison of the changes observed at ambient temperature with Δ*t* = 10 ms obtained in the present study, with those of the previous study, where the S_2_ state was trapped cryogenically with Δ*t* ≃ 1 s and the diffraction data collected at low temperature (Suga *et al.*, 2019 ▸), as well as that carried out at ambient temperature with Δ*t* = 200 ms (Kern *et al.*, 2018 ▸), shows an overall tendency of the structural changes. The changes found in the OEC were identical regardless of the temperature or the different Δ*t*, suggesting the stability and long lifetime of the catalytic center in the S_2_ state. By contrast, the changes found in some amino acid residues and water molecules differ substantially either depending on the temperatures or Δ*t*, reflecting a rather large mobility of the protein environment. This tendency is apparent in the Q_B_ site, in which the quinone changes its redox forms during the catalytic cycle. The changes in the Q_B_ site were larger with Δ*t* = 10 ms but smaller with Δ*t* = 200 ms (Kern *et al.*, 2018 ▸) and further diminished at Δ*t* ≃ 1 s (Suga *et al.*, 2019 ▸). However, the Q_B_ yield based on the oxidation status of the non-heme iron may be different under experimental conditions.

Comparisons of the structural changes in the O1 and O4 channels with previous crystallographic studies (Suga *et al.*, 2019 ▸; Kern *et al.*, 2018 ▸) also reveal the differences in the channels as a function of the S*_i_*-state. W665, the second water from O4 in the O4 channel, became highly mobile in the S_1_-to-S_2_ transition, which is consistent with previous studies. The disorder of W665 breaks the hydrogen-bonded network in the O4 channel in the S_2_ state. In our initial report of the disorder of W665 in the S_2_ state, we proposed that the proton transfer may occur in the O4 channel in the S_1_-to-S_2_ transition (Suga *et al.*, 2019 ▸). The changes observed in the O4 channel were similar to the theoretical calculation that reported a proton release in the channel in the S_0_-to-S_1_ transition (Saito *et al.*, 2015 ▸). However, FTIR studies on the D1-S169A mutant, which perturbs the interaction of a water molecule hydrogen bonded to O4, showed only minor effects on the efficiencies or kinetics of the S*_i_*-state transitions (Shimada *et al.*, 2020 ▸; Ghosh *et al.*, 2019 ▸). In contrast, the S_2_ state multiline EPR spectrum of the D1-S169A mutant differs significantly from that of wild-type (Ghosh *et al.*, 2019 ▸). A further theoretical study on the O4 channel showed that removing water molecules in the O4 channel results in a decrease in the S_2_/S_1_ redox potential by ∼80 mV (Mandal *et al.*, 2020 ▸). Therefore, the weakened hydrogen-bonded network resulting from the increased mobility of W665 may be necessary for the decrease in the redox potential to facilitate the S_1_-to-S_2_ transition.

In contrast to the changes observed in the O4 channel, the changes that occurred in the water molecules of the O1 channel differ between the present and previous studies. W601, the hydrogen-bond donor to O1, became disordered in the present study at Δ*t* = 10 ms, and two additional water molecules W547 and W536 (W26, W27 and W29) (Kern *et al.*, 2018 ▸) became disordered at Δ*t* = 200 ms. However, instead of these water molecules, W571 became disordered in the study with Δ*t* ≃ 1 s at cryogenic temperature (Suga *et al.*, 2019 ▸). Because the O1 channel is a broad channel where water molecules are the major component of the hydrogen-bonded network, substrate water molecules are likely to propagate through the water molecules in the O1 channel. The differences observed in the structural changes of water molecules located in this channel at different temperatures and/or delay times after laser flash illumination may reflect the high mobility of water molecules in this channel. Here, we could not exclude the possibility that the structural differences were caused by the difference in the solution conditions in which the crystals were made and/or kept. It should be noted that glycerol or DMSO molecules used as cryo-protectants were indeed identified in the O1 channel (Umena *et al.*, 2011 ▸; Suga *et al.*, 2015 ▸), suggesting the entry of outside molecules into this channel. Thus, increasing the mobility of water in this channel seems beneficial for driving the incorporation of substrate water into the catalytic site in the following S_3_ state.

**Figure 7:**
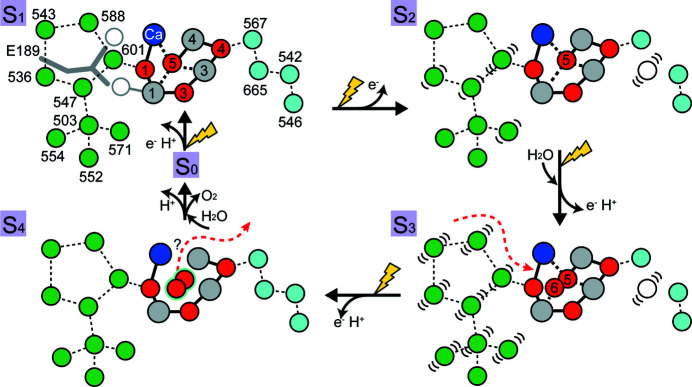
Structural changes in the OEC and water molecules found in the present and previous studies are summarized. Red and gray spheres with numbers in the OEC are oxygen and manganese atoms, respectively, and the Mn2 and O2 atoms have been omitted for clarity.

Fig. 7 ▸ summarizes the structural changes regarding the water molecules in the O1 and O4 channels observed in the present study. In the S_1_-to-S_2_ transition, W665 in the O4 channel becomes highly mobile, disconnecting the 15 Å-long water chain starting from O4-W567. W665 remains disordered in the S_3_ state but becomes re-ordered beyond the S_3_ state (Suga *et al.*, 2019 ▸; Kern *et al.*, 2018 ▸). W601 in the O1 channel also becomes disordered in the S_1_-to-S_2_ transition. The mobility of W601 is propagated to W547, W536 and W571 through the hydrogen-bonded network in the O1 channel. D1-E189, the only monodentate carboxyl ligand of the OEC, flips by 0.5 Å in the following S_2_-to-S_3_ transition, leading to broadening of the O1 channel, thereby further increasing the mobility of water molecules there (Suga *et al.*, 2017 ▸, 2019 ▸; Ibrahim *et al.*, 2020 ▸). The flipping of D1-E189 makes a space in the vicinity of O5 to allow the insertion of the additional water (oxygen) O6 in the subsequent S_2_-to-S_3_ transition. This suggests that water molecules in the O1 channel may be the source for O6. Di­oxy­gen is then probably formed between O5 and O6 by an oxyl/oxo coupling mechanism, or between O5 and another water molecule, and in this case, O6 refills the empty substrate site. The changes of water molecules in the O1 channel support the mechanism of water delivery from the Ca side (Kim & Debus, 2019 ▸; Sakamoto *et al.*, 2017 ▸; Suga *et al.*, 2019 ▸), rather than the O4 channel (Kawashima *et al.*, 2018 ▸; Wang *et al.*, 2017 ▸; Retegan *et al.*, 2016 ▸). Further structural analysis will be required to reveal how the OEC incorporates those water molecules in the O1 channel and how protons egress during the subsequent S-state transitions.

## Supplementary Material

Supporting information file. DOI: 10.1107/S2052252521002177/zf5015sup1.pdf

PDB reference: dark1, 7cou

PDB reference: dark2, 7cji

PDB reference: 10 ms, light, S_2_ state, 7cjj

## Figures and Tables

**Table 1:** Statistics for data collection and structure refinement Values in parentheses are those of the highest resolution shell. Structure factors for −50 ns, light datasets at flow rates of 4.9 µl min^−1^ (Asf), 7.3 µl min^−1^ (Bsf), 8.5 µl min^−1^ (Csf), and 9.8 µl min^−1^ (Dsf) have been deposited in the Protein Data Bank (PDB) with the accession number 7cou.

Data name	Dark1	Dark2	−50 ns, light, 4.9 µl min^−1^	−50 ns, light, 7.3 µl min^−1^	−50 ns, light, 8.5 µl min^−1^	−50 ns, light, 9.8 µl min^−1^	10 ms, light, 9.8 µl min^−1^
Data collection							
Flow rate (µl min^−1^)	2.5	2.5	4.9	7.3	8.5	9.8	9.8
No. of indexed images	96459	41071	18216	13858	20449	22419	17247
Space group	*P*2_1_2_1_2_1_	*P*2_1_2_1_2_1_	*P*2_1_2_1_2_1_	*P*2_1_2_1_2_1_	*P*2_1_2_1_2_1_	*P*2_1_2_1_2_1_	*P*2_1_2_1_2_1_
Unit cell parameters (Å)	*a* = 126.1	*a* = 126.0	*a* = 125.8	*a* = 126.0	*a* = 125.7	*a* = 125.6	*a* = 125.7
	*b* = 231.7	*b* = 231.7	*b* = 231.5	*b* = 231.6	*b* = 231.6	*b* = 231.7	*b* = 231.5
	*c* = 288.3	*c* = 288.3	*c* = 288.2	*c* = 288.2	*c* = 288.4	*c* = 288.5	*c* = 288.3
Resolution (Å)	40–2.25	40–2.35	40–2.40	40–2.40	40–2.40	40–2.40	40–2.40
Highest shell (Å)	2.33–2.25	2.43–2.35	2.49–2.40	2.49–2.40	2.49–2.40	2.49–2.40	2.49–2.40
No. of unique reflections	399661	351162	329839	329852	329843	329840	329844
Completeness (%)	100	100	100	100	100	100	100
Multiplicity	847 (547)	494 (339)	159 (109)	111 (76)	249 (172)	271 (187)	235 (162)
*R*_split_ (%)	4.6 (52.4)	5.7 (56.5)	8.1 (60.7)	10.8 (76.6)	6.7 (53.3)	7.3 (56.4)	8.4 (63.8)
CC_1/2_	0.999 (0.62)	0.999 (0.60)	0.996 (0.610)	0.994 (0.452)	0.998 (0.706)	0.997 (0.643)	0.997 (0.540)
Mean *I*/σ(*I*)	65.3 (2.2)	56.8 (2.0)	36.9 (1.8)	27.1 (1.4)	43.1 (2.1)	40.2 (2.0)	37.0 (1.8)
							
Refinement							
*R*_work_/*R*_free_ (%)	0.169/0.211	0.160/0.208	–	–	–	–	0.168/0.219
Wilson *B* (Å^2^)	44.0	48.4	–	–	–	–	46.1
Average *B* factor (Å^2^)	61.3	67.1	–	–	–	–	63.1
Protein	62.2	67.0	–	–	–	–	63.0
OEC	43.2	51.2	–	–	–	–	48.1
Water	64.0	69.1	–	–	–	–	66.0
RMSD bond length (Å)	0.008	0.008	–	–	–	–	0.008
RMSD bond angle (°)	1.238	1.245	–	–	–	–	1.263
Ramachandran (%)							
Favoured	97.71	97.64	–	–	–	–	97.66
Allowed	2.11	2.25	–	–	–	–	2.21
Outliers	0.17	0.12	–	–	–	–	0.13
PDB entry	7cou	7cji	7cou	7cou	7cou	7cou	7cjj

**Table 2:** Interatomic distances of the OEC and their comparisons between different structures Values are averages of the nominal distances in two non-crystallographic symmetry related monomers.

	The present study (room temperature)		100 K (Suga *et al.*, 2019 ▸)		Room temperature (Kern *et al.*, 2018 ▸)	
	S_1_	S_2_	S_1_	S_2_	S_1_	S_2_
Mn1–Mn2	2.66	2.64	2.60	2.68	2.78	2.81
Mn1–Mn3	3.16	3.15	3.16	3.21	3.25	3.26
Mn1–Mn4	4.94	4.90	4.97	4.90	4.86	4.86
Mn2–Mn3	2.76	2.72	2.72	2.75	2.85	2.84
Mn2–Mn4	5.28	5.26	5.27	5.20	5.21	5.24
Mn3–Mn4	2.86	2.84	2.89	2.76	2.74	2.74
Mn1–Ca	3.58	3.53	3.61	3.51	3.43	3.42
Mn2–Ca	3.44	3.44	3.42	3.40	3.38	3.41
Mn3–Ca	3.49	3.56	3.40	3.46	3.51	3.52
Mn4–Ca	3.90	4.04	3.76	3.90	3.83	3.90
Mn1–Mn2	2.66	2.64	2.60	2.68	2.78	2.81
Mn1–Mn3	3.16	3.15	3.16	3.21	3.25	3.26
Mn1–Mn4	4.94	4.90	4.97	4.90	4.86	4.86
