# Supplementary material for: Capturing structural changes of the S1 to S2 transition of photosystem II using time-resolved serial femtosecond crystallography
Source: IUCrJ. 2021 Apr 7;8(Pt 3):431–43. doi: 10.1107/S2052252521002177 (PMC8086164; doi:10.1107/S2052252521002177)
Supplement: Supplementary file 1 [file m-08-00431-sup1.pdf]

IUCrJ

Volume 8 (2021)

Supporting information for article:

Capturing structural changes of the S1 to S2 transition of photosystem II using time-resolved serial femtosecond crystallography

Hongjie Li, Yoshiki Nakajima, Takashi Nomura, Michihiro Sugahara, Shinichiro Yonekura, Siu Kit Chan, Takanori Nakane, Takahiro Yamane, Yasufumi Umena, Mamoru Suzuki, Tetsuya Masuda, Taiki Motomura, Hisashi Naitow, Yoshinori Matsuura, Tetsunari Kimura, Kensuke Tono, Shigeki Owada, Yasumasa Joti, Rie Tanaka, Eriko Nango, Fusamichi Akita, Minoru Kubo, So Iwata, Jian-Ren Shen and Michihiro Suga

Table S1 List of peak heights in the isomorphous difference maps.

	Absolute peak value of W665 (σ)			Absolute peak value of W601 (σ)			Maximum noise (σ)	1σ ($e^-/Å^3$)
	A	B	Average	A	B	Average		
-50 ns, “light”, 4.9 μl/min	7.4	6.3	6.9	6.3	4.1	5.2	4.2	0.018
-50 ns, “light”, 7.3 μl/min	4.3	2.9	3.6	3.4	3.2	3.3	4.5	0.018
-50 ns, “light”, 8.5 μl/min	5.4	3.5	4.5	1.8	2.7	2.3	4.1	0.019
-50 ns, “light”, 9.8 μl/min	3.6	3.2	3.4	1.8	1.5	1.7	4.1	0.022
10 ms, light, 9.8 μl/min	12.4	10.8	11.6	7.4	7.3	7.4	4.3	0.019

A and B denote A- and B- monomers in the dimeric photosystem II.
